# Prevalence and incidence of overweight and obesity among Vietnamese preschool children: a longitudinal cohort study

**DOI:** 10.1186/s12887-017-0904-y

**Published:** 2017-06-19

**Authors:** Loan Minh Do, Toan Khanh Tran, Bo Eriksson, Max Petzold, Henry Ascher

**Affiliations:** 1 Outpatient Department, National Hospital of Paediatrics, 18/879 La Thanh Road, Dong Da District, Hanoi, Vietnam; 2 Family Medicine Department, Hanoi Medical University, No.1 Ton That Tung Street, Hanoi, Vietnam; 3 Health Metrics, Department of Public Health and Community Medicine, Institute of Medicine at the Sahlgrenska Academy, University of Gothenburg, PO Box 440, -405 30 Gothenburg, SE Sweden; 4 School of Public Health, Faculty of Health Sciences, University of the Witwatersrand, Johannesburg, South Africa; 5 Section for Epidemiology and Social Medicine (EPSO), Department of Public Health and Community Medicine, Institute of Medicine at the Sahlgrenska Academy, University of Gothenburg, PO Box 453, -405 30 Gothenburg, SE Sweden; 6 Angered Hospital, Gothenburg, Sweden

**Keywords:** Overweight, Obesity, Preschool children, Longitudinal study, Vietnam

## Abstract

**Background:**

A plateau in childhood overweight and obesity has been reported in some developed countries while in almost all developing countries this problem is on the rise. The aim of this paper is to describe the changes in prevalence of overweight and obesity within a cohort of preschool children followed for 3 years, and to estimate and compare the incidences in urban and rural children of Hanoi, Vietnam.

**Methods:**

A longitudinal study of a cohort of 2677 children aged 3 to 6 years old at the beginning of the study was conducted in urban DodaLab and rural FilaBavi, Hanoi, Vietnam. Overall, 2602 children, 1311 urban and 1291 rural, were followed for 3 years with identical measurements of weight and height in 2013, 2014 and 2016. Standard methods were used to estimate prevalence and incidence as well as confidence intervals.

**Results:**

During the three-year follow-up, the overall estimated prevalence of overweight increased from 9.1% to 16.7%. For the urban children, the increase was considerably higher. The overall prevalence of obesity decreased from 6.4% to 4.5% with less decrease in the urban children. In the group of children who were overweight and obese at the start of the study, 41.4% and 30.7%, respectively, remained in the same state three years later. The incidence of overweight and obesity during the three years were 12.4% and 2.7%, respectively. Boys were more likely to develop obesity than girls.

**Conclusions:**

Already in preschool age, the prevalence of overweight is high and it continues to increase with age, especially in the urban area. Prevention and intervention programs need to start at early preschool age and actions in urban areas deserve priority.

## Background

Childhood overweight and obesity are conditions traditionally thought to primarily occur in countries with a high level of social and economic resources. In high-income countries the prevalence of overweight and obesity has increased dramatically since the 1980s [1–3]. However, during the past three decades it has spread throughout the world [4, 5]. Recent studies have shown a stabilization of this prevalence over the last 10 years, at least in some developed countries [6] like USA [7], Australia [8], England, France, Sweden, Switzerland and Poland [9, 10]. A study in Italy even found a decrease in prevalence of overweight including obesity in Tuscan children from 32% in 2002 to 25.8% in 2012 [11].

The prevalence of childhood overweight and obesity remains low in developing countries compared to developed countries, particularly in Asia [12]. In the 1990s, the globally estimated prevalence of obesity in children in low-income countries was 3% [3]. However, some recent results show changing trends: the rate of increase for overweight and obesity in low- and middle-income countries may be even greater than in high-income countries [13, 14].

In Vietnam, which recently became a middle-income country, childhood overweight and obesity were not reported as a problem before 1995 [15]. The Doi Moi (new policy) reform in 1986 marked a significant turning point in the transformation of the Vietnamese economy into an open, market-oriented and globally integrated model. Since the reform, the Vietnamese economy has grown rather quickly [16]. This has contributed to dietary and lifestyle changes that encourage the consumption of high-energy foods, including processed foods and food consumed outside the home. This, combined with reduced energy expenditure from increased sedentary behavior [17], has resulted in rising problems of overweight: the estimated prevalence of overweight in Vietnamese children under 5 years has increased from 1.4% in 1998 to 2.6% in 2005 and 4.6% in 2010 [18]. Studies in big cities show an increasing trend. For example, a longitudinal study of adolescents in urban districts of Ho Chi Minh City between 2004 and 2009 indicated that the prevalence of overweight and obesity increased gradually from 12.5% and 1.7%, respectively, in the first year to 16.7% and 5.1% in the last year [19].

The aim of this paper is to describe the changes in prevalence of overweight and obesity within a cohort of preschool children followed for 3 years, and to estimate and compare the incidences of overweight and obesity in urban and rural children of Hanoi, Vietnam.

## Methods

### Study design and setting

The full study consists of a baseline survey in 2013, reported in a previous paper [20], and a three-year follow-up thereafter of the participating children with identically repeated surveys in 2014 and 2016.

The study was conducted in two Health and Demographic Surveillance Sites (HDSS), urban DodaLab [21] and rural FilaBavi [22], both located in Hanoi, Vietnam. The sites were established for different purposes including serving as a background and sampling frame for specific studies. Information about demographic and socioeconomic characteristics of households and individuals has been collected every 2 years. The information about vital events such as birth, death and migration has been updated quarterly [21, 22].

### Participating children

The study included children who were 3 to 6 years old at the time of the first survey in 2013. The HDSS database in 2012 was used to select participants. Children with certain diagnosed medical conditions affecting growth, such as hypothyroidism, Cushing disease et cetera or children who had taken corticosteroids in the past 6 months were excluded from the study. At the start of the study 2842 children, 1482 in the urban site and 1360 in the rural site, living in strategically selected communes were recruited. Of those, 2677 children, 1364 urban and 1313 rural, participated in the first survey in 2013 [20]. At the end, in the third survey, complete weight and height information were obtained from 2602 children, 1311 urban and 1291 rural children. The total number of children who dropped out was 75 (2.8%); 53 (3.9%) from the urban site and 22 (1.7%) from the rural sites. Of these 75, 7 children (9.3%) were overweight and 5 (6.7%) were obese. The reason for withdrawal from the study was that the families moved to other places and could not be reached. Only those who participated in all three surveys were used in the analyses.

### Measurement and data sources

The data used in the study included general information about child and parent characteristics, demographic and socioeconomic status of the family, specific information for the present study and anthropometric measurements. This information was obtained by interviewing parents or caregivers using a structured questionnaire specifically designed for the study. The questionnaire consisted of 54 questions: 33 questions about the child and 21 questions about the family. Weight and height of the children were measured in the homes by well-trained staff working in pairs [20]. Digital Tanita scales and mobile measurements were used as instruments. Measurements were made to the nearest 0.1 kg and 0.1 cm respectively.

The interview procedure and measurement quality control was the same in all the surveys, 2013, 2014 and 2016 [20]. Eight field workers in DodaLab and 12 in FilaBavi were responsible for interviewing and taking anthropometric measurements of children. The aim of the training for interviewers was that all field work should be done exactly in the same way in the urban and rural areas throughout the entire timeframe, to make them comfortable with the questions and find a suitable language in the interview without changing the meaning. The interviewers were trained to accurately measure weight and height. The same field workers were involved in the baseline and follow-up studies to minimize measurement errors. The data quality control was performed by supervisors and the researcher by checking randomly about 3% of the anthropometric measurements and records. A correction was needed in only three cases.

### Study size

The number of children from each site, urban and rural, was originally decided to be about 1300, that is totally 2600. It was assumed that any prevalence observed in the study would not exceed 20% and the ambition was set to have confidence intervals with length not exceeding 2.5% units in estimated prevalence for an individual site. For practical reasons, the total number, 2677, became slightly larger.

### Statistical analysis

The children were classified as overweight or obese according to the definitions of International Obesity Task Force (IOTF) [23]. Body Mass Index (BMI), calculated in the traditional way as BMI = weight/(height)^2^ (weight in kg, height in m).

Age sub-cohorts, 3, 4, 5 or 6 years, were defined according to the age of the child at the time of the first survey in 2013. The changes over time were evaluated for statistical significance using differences.

All estimation and testing was performed using Stata software, version 14. Estimation of prevalences and incidences and their confidence limits as well as statistical tests were done using standard statistical methods. Normal approximation confidence limits and tests were used when considered feasible. In other situations with small numbers of observations and cases, exact probability options were used.

### Ethical considerations

The field sites, FilaBavi and DodaLab, have been ethically approved by the Ministry of Health of Vietnam as well as by the Scientific and Ethical Committee of Hanoi Medical University. Permissions have also been granted by Dong Da and Ba Vi district authorities. In addition, the Scientific and Ethical Committee of Hanoi Medical University has specifically approved this research of overweight and obesity. Verbal consent from all caregivers of participating children in the study was obtained.

## Results

The study population is described in Table 1. The numbers of urban and rural children in the study were roughly equal and about the same numbers of boys and girls participated in the study. The children who dropped out of the study were randomly spread over the age sub-cohorts.

**Table 1:** Distribution of the children participating in the baseline survey (2013) and in the two subsequent surveys (2014 and 2016)

		2013 (*n* = 2677)	2014 (*n* = 2618)	2016 (*n* = 2602)
Area of residence	Urban; n (%)	1364 (50.9)	1321 (50.5)	1311 (50.4)
	Rural; n (%)	1313 (49.1)	1297 (49.5)	1291 (49.6)
Sex	Boys; n (%)	1430 (53.4)	1398 (53.4)	1391 (53.5)
	Girls; n (%)	1247 (46.6)	1220 (46.6)	1211 (46.5)
Age sub-cohort	3 years; n (%)	765 (28.6)	751 (28.7)	745 (28.6)
	4 years; n (%)	875 (32.7)	859 (32.8)	854 (32.8)
	5 years; n (%)	886 (33.1)	860 (32.8)	857 (32.9)
	6 years; n (%)	151 (5.6)	148 (5.7)	146 (5.6)

The means BMI of children in the different age sub-cohorts for the three surveys are shown in Table 2. The mean BMI of urban children was higher than for the rural and above the WHO median curve in all age sub-cohorts. In contrast, the mean BMI of rural children for almost all age sub-cohorts were below the WHO median curve. The difference in mean BMI between the 2016 and 2013 surveys was positive for all urban groups and negative for the rural, except the children in age sub-cohort 6 and the rural boys in age sub-cohort 5.

**Table 2:** Estimated mean BMI for children by urban/rural, boys/girls and age sub-cohorts

	Age sub-cohort	Number of children	2013	2014	2016	Difference (2016–2013)
Urban total		1311	15.9	16.1	17.1	1.2
Urban boys	Total	699	16.1	16.3	17.3	1.2
	3 years	169	15.9	16.0	16.8	0.9
	4 years	242	15.7	16.1	17.1	1.4
	5 years	232	16.6	16.8	17.9	1.3
	6 years	56	16.1	16.3	17.3	1.2
Urban girls	Total	612	15.7	15.8	16.9	1.2
	3 years	140	15.7	15.8	16.8	1.1
	4 years	197	15.8	15.8	16.9	1.1
	5 years	228	15.8	15.9	16.9	1.1
	6 years	47	15.3	15.9	16.7	1.4
Rural total		1291	15.4	14.0	15.2	−0.2
Rural boys	Total	692	15.4	15.0	15.3	−0.1
	3 years	245	15.7	15.2	15.3	−0.4
	4 years	219	15.2	14.8	15.0	−0.2
	5 years	204	15.3	15.0	15.7	0.4
	6 years	24	14.4	14.6	15.3	0.9
Rural girls	Total	599	15.3	14.8	15.0	−0.3
	3 years	191	15.5	15.1	14.8	−0.7
	4 years	196	15.3	14.7	15.0	−0.3
	5 years	193	15.2	14.8	15.1	−0.1
	6 years	19	15.0	14.3	14.9	0.1

### Overweight during three-year follow-up

**Fig. 1:**
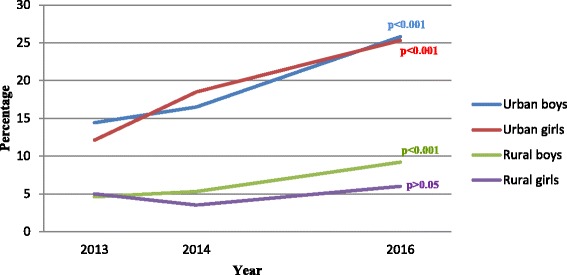
Estimated prevalence of overweight by area and sex. *(p-values refer to the comparison between estimates for 2013 and 2016)*

The estimated prevalence of overweight (obesity not included) by area, sex and age sub-cohorts are shown in Table 3 and Fig. 1. The prevalence of overweight for all children who participated in the three surveys increased from 9.1% in 2013 to 16.7% in 2016. The prevalence as well as the rate of increase was consistently higher in the urban children than in the rural. The former almost doubled, from 13.3% in the first survey to 25.6% in the last. The corresponding prevalence among rural children increased from 4.8% to 7.7%.

**Table 3:** Estimated prevalence of overweight by area, sex and age sub-cohort during the follow-up from 2013 to 2016

	Age sub-cohort	Number of children	Baseline % 95% CI	One year older % 95% CI	Three years older % 95% CI	Difference (2016–2013)
All children		2602	9.1 8.0–10.2	11.0 9.8–12.2	16.7 15.3–18.2	7.6^b^***
Urban total		1311	13.3^a^*** 11.5–15.2	17.4^a^*** 15.3–19.4	25.6^a^*** 23.2–27.9	12.3^b^***
Urban boys	3 years	169	7.1 3.2–11.0	10.1 5.5–14.6	22.5 16.1–28.8	15.4^b^***
	4 years	242	12.0 7.9–16.1	16.5 11.8–21.2	23.6 18.2–28.9	11.6^b^***
	5 years	232	21.6 16.2–26.9	19.8 14.7–25.0	32.8 26.7–38.8	11.2^b^**
	6 years	56	17.9 7.5–28.2	21.4 10.3–32.5	16.1 6.1–26.0	−1.8
Urban girls	3 years	140	12.1 6.7–17.6	17.9 11.4–24.3	32.1 24.3–40.0	20.0^b^***
	4 years	197	9.6 5.5–13.8	19.3 13.7–24.8	28.4 22.1–34.8	18.8^b^***
	5 years	228	14.5 9.9–19.1	19.3 14.1–24.5	18.9 13.7–24.0	4.4
	6 years	47	10.6 1.5–19.8	12.8 2.9–22.7	23.4 10.8–36.0	12.8
Rural total		1291	4.8 3.6–6.0	4.5 3.4–5.6	7.7 6.3–9.2	2.9^b^**
Rural boys	3 years	245	4.5 1.9–7.1	6.1 3.1–9.1	11.4 7.4–15.4	6.9^b^**
	4 years	219	3.2 0.8–5.5	5.0 2.1–7.9	7.3 3.8–10.8	4.1^b^*
	5 years	204	6.9 3.4–10.4	5.4 2.3–8.5	9.3 5.3–13.3	2.4
	6 years	24	0.0 0.0–14.2	0.0 0.0–14.2	4.2 0.0–21.1	4.2
Rural girls	3 years	191	4.2 1.3–7.1	4.2 1.3–7.1	5.2 2.0–8.4	1.0
	4 years	196	4.6 1.6–7.5	2.6 0.3–4.8	6.6 3.1–10.1	2.0
	5 years	193	6.2 2.8–9.7	4.1 1.3–7.0	6.2 2.8–9.7	0.0
	6 years	19	5.3 0.1–26.0	0.0 0.0–17.6	5.3 0.1–26.0	0.0

^a^ *Urban* vs. *rural*

^b^ *2016* vs. *2013*

*p < 0.05

**p < 0.01

***p < 0.001

No statistically significant difference in the prevalence of overweight was found between boys and girls except that rural boys were more likely to be overweight than rural girls in the last survey.

A similar pattern of increasing overweight was seen in almost all age sub-cohorts although not all were statistically significant.

### Obesity during three-year follow-up

The overall prevalence of obesity decreased in the second survey and there were only small changes in the third survey for both urban and rural children (Table 4). In urban children, obesity decreased from 9.2% to 7.1% and in rural from 3.5% to 1.9%. A tendency of a generally higher prevalence of obesity in the urban than in the rural children was found for the whole study period.

**Table 4:** Estimated prevalence of obesity by area, sex and age sub-cohort during the follow-up from 2013 to 2016

	Age sub-cohort	Number of children	Baseline % 95% CI	One year older % 95% CI	Three years older % 95% CI	Difference (2016–2013)
All samples		2602	6.4 5.4–7.3	4.4 3.6–5.2	4.5 3.7–5.3	−1.9^b^**
Urban total		1311	9.2^a^*** 7.7–10.8	7.5^a^*** 6.1–8.9	7.1^a^*** 5.7–8.5	−2.1^b^*
Urban boys	3 years	169	8.9 4.5–13.2	8.3 4.1–12.5	7.7 3.6–11.8	−1.2
	4 years	242	7.0 3.8–10.3	7.9 4.4–11.3	10.3 6.5–14.2	3.3
	5 years	232	15.5 10.8–20.2	11.6 7.5–15.8	9.5 5.7–13.3	−6.0
	6 years	56	7.1 0.2–14.1	5.4 1.1–14.9	3.6 0.4–12.3	−3.5
Urban girls	3 years	140	4.3 0.9–7.7	6.4 2.3–10.5	8.6 3.9–12.3	4.3
	4 years	197	12.7 8.0–17.4	7.1 3.5–10.7	5.6 2.3–8.8	−7.1^b^*
	5 years	228	7.5 4.0–10.9	4.8 2.0–7.6	3.5 1.1–5.9	−4.0
	6 years	47	2.1 0.1–11.3	2.1 0.1–11.3	0.0 0.0–7.5	−2.1
Rural total		1291	3.5 2.5–4.5	1.3 0.7–1.9	1.9 1.1–2.6	−1.6^b^*
Rural boys	3 years	245	2.9 0.8–5.0	1.6 0.03–3.2	4.9 2.2–7.6	2.0
	4 years	219	2.7 0.6–4.9	0.5 0.0–2.5	0.5 0.0–2.5	−2.2
	5 years	204	3.9 1.2–6.6	1.5 0.3–4.3	1.0 0.1–3.5	−2.9
	6 years	24	0.0 0.0–14.2	0.0 0.0–14.2	0.0 0.0–14.2	0.0
Rural girls	3 years	191	3.1 0.6–5.6	2.1 0.05–4.1	3.7 1.0–6.3	0.6
	4 years	196	4.1 1.3–6.9	2.0 0.04–4.0	0.5 0.0–2.8	−3.6^b^*
	5 years	193	4.7 1.7–7.7	0.5 0.0–2.9	0.5 0.0–2.9	−4.2^b^**
	6 years	19	5.3 0.1–26.0	0.0 0.0–17.6	0.0 0.0–17.6	−5.3

^a^ *Urban* vs. *rural*

^b^ *2016* vs. *2013*

*p < 0.05

**p < 0.01

***p < 0.001

**Fig. 2:**
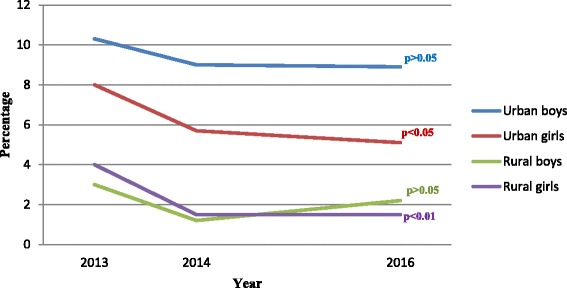
Estimated prevalence of obesity by area and sex. *(p-values refer to the comparison between estimates for 2013 and 2016)*

Looking at sex differences, the observed pattern of decreasing obesity was not found for urban and rural boys where the prevalence did not change significantly within the three years (Fig. 2). There were no statistically significant differences in the prevalence of obesity between rural boys and rural girls. However, urban boys were more likely to be obese than urban girls in the second and third surveys.

A decrease was seen in obesity prevalence for most age sub-cohorts. For the youngest urban girls and the rural children the prevalence instead increased. However, almost all these changes were not statistically significant possibly due to the relatively small numbers of cases.

### Incidence of overweight and obesity

Table 5 summarizes the individual changes in overweight and obesity over the three years. For the whole study population, the percentages of children who were overweight and obese in 2013 and remained in the same state in 2016 were 41.4% and 30.7% respectively. The table also shows estimates of incidences in which 12.4% and 2.7% children developed overweight and obesity between the 2013 and the 2016 surveys, respectively.

**Table 5:** Estimated percentages of children remaining overweight or obese together with estimated incidences from 2013 to 2016

	Age sub-cohort	Remaining Ow or Ob in 2016		Incidence	
		Overweight % (number of Ow children in 2013)	Obesity % (number of Ob children in 2013)	Overweight % (number of non-Ow and non-Ob children in 2013)	Obesity % (number of non-Ob children in 2013)
All children		41.4 (237)	30.7 (166)	12.4 (2199)	2.7 (2436)
Urban total		46.3^a^**(175)	36.4^a^**(121)	19.9^a^***(1015)	4.1^a^***(1190)
Urban boys	All	49.5 (101)	38.9 (72)	19.8 (526)	5.4^b^* (627)
	3 years	41.7 (12)	26.7 (15)	19.0 (142)	5.8 (154)
	4 years	48.3 (29)	70.6 (17)	20.9 (196)	5.8 (225)
	5 years	52.0 (50)	30.6 (36)	22.6 (146)	5.6 (196)
	6 years	50.0 (10)	25.0 (4)	7.1 (42)	1.9 (52)
Urban girls	All	41.9 (74)	32.7 (49)	20.0 (489)	2.7 (563)
	3 years	41.2 (17)	66.7 (6)	31.6 (117)	6.0 (134)
	4 years	63.2 (19)	28.0 (25)	20.3 (153)	2.3 (172)
	5 years	33.3 (33)	29.4 (17)	11.8 (178)	1.4 (211)
	6 years	20.0 (5)	0.0 (1)	22.0 (41)	0.0 (46)
Rural total		27.4 (62)	15.6 (45)	5.9 (1184)	1.4 (1246)
Rural boys	All	37.5 (32)	19.1 (21)	6.7 (639)	1.6 (671)
	3 years	27.3 (11)	14.3 (7)	8.8 (227)	4.6 (238)
	4 years	14.3 (7)	16.7 (6)	6.3 (206)	0.0 (213)
	5 years	57.1 (14)	25.0 (8)	5.0 (182)	0.0 (196)
	6 years	0.0 (0)	0.0 (0)	4.2 (24)	0.0 (24)
Rural girls	All	16.7 (30)	12.5 (24)	5.0 (545)	1.0 (575)
	3 years	12.5 (8)	50.0 (6)	3.4 (177)	2.2 (185)
	4 years	11.1 (9)	0.0 (8)	6.2 (179)	0.5 (188)
	5 years	25.0 (12)	0.0 (9)	5.2 (172)	0.5 (184)
	6 years	0.0 (1)	0.0 (1)	5.9 (17)	0.0 (18)

*Ow overweight; Ob Obesity*

*ªUrban* vs. *rural*

^b^ *Urban boys* vs. *urban girls*

*p < 0.05

**p < 0.01

***p < 0.001

The urban children were more likely to stay overweight than the rural children (46.3% vs. 24.7%), and were three times more likely than rural children to develop overweight during the study period. A similar pattern was found for obesity: 36.4% and 15.6% of the obese children remained obese in the urban and the rural areas, respectively. Among the urban children, 4.1% developed obesity during the study period, while only 1.4% of rural children did so.

There was a non-significant tendency that more boys than girls remained overweight and obese and the difference in overweight incidence was still smaller. However, the incidence of obesity for boys was about double compared to girls in the urban area.

The differences between age sub-cohorts in remaining or developing overweight and obesity varied without any distinct pattern.

## Discussion

This longitudinal cohort study allows the study of individual changes in child’s weight status. Over the three years of follow-up, within the cohort, there was a remarkable increase in overweight prevalence among the urban children, a smaller increase in rural, and a slight decrease in obesity both in urban and rural children. The incidence of overweight, higher in urban children than in the rural, was much higher than the incidence of obesity.

The results of the present study are consistent with cross-sectional studies done in Vietnam showing that overweight and obesity among children are more prevalent in urban and wealthy areas than in the rural and less wealthy areas [24–26]. The findings indicate that patterns of overweight and obesity prevalence within the cohort were similar in urban and rural areas. The development of overweight and obesity in children could be influenced by characteristics of individual, family, community and society at large which interact with each other [27]. Urban and rural areas in Vietnam are significantly different in most of these characteristics. For example, in urban areas processed food restaurants are more available, game shops are more common and spaces for play and leisure are more limited than in rural areas. The differences in community characteristics could be one of the explanations of the differences in overweight and obesity prevalence between the two areas in this study. Living in Vietnam, both in urban and rural areas, is influenced by the same traditions, culture, governmental policies as well as economic development. There is, though, much variation in individual responses. National level influences could to some extent explain similarities in overweight and obesity development between the urban and the rural children.

In this study, the prevalence of combined overweight and obesity among preschool children in the urban area of Hanoi defined by IOTF cut-off values was 22.6% in 2013. Cross-sectional studies on preschool age children (3–6 years) in the urban area of Ho Chi Minh City (HCMC) using the same definition showed that the prevalence increased from 21.4% in 2002 to 36.8% in 2005 [28]. Ho Chi Minh City, Vietnam’s largest city, located in the south, is the economic center of the country. Hanoi, the second largest city, located in the north, is the capital and political center. Although sharing some common Vietnamese culture and custom, the two cities are different in economy and environment as well as lifestyle and eating habits. For example, in 2015 HCMC’s GDP grew 9.8%, with GDP per capita reaching USD$5500 [29]. In the same period, yearly income per capita in Hanoi was about USD$3600 [30]. HCMC citizens have, to a higher extent, been influenced by American lifestyle and eating habits, as it was occupied by USA for about 20 years. This could be an explanation for the slower and later development of childhood overweight and obesity in Hanoi.

The upward tendency of overweight including obesity with a higher prevalence and a faster rate of development in urban areas than in rural areas might reflect involvement of environmental factors contributing to the increase. It also suggests that special attention is needed for prevention and management of childhood overweight and obesity in urban areas and big cities.

The childhood overweight and obesity epidemic started early in developed countries. There is now an awareness of the consequences and many prevention programs focusing on modifying lifestyle and eating habits have been designed [31, 32]. The presently observed stability in obesity prevalence could be the results of these accumulated actions [33]. To prevent childhood overweight and obesity developing countries, including Vietnam, need to learn from these experiences. If action is taken immediately, it should be possible to limit the increasing trend.

In the present study, the trends in prevalence of overweight and obesity are similar for boys and girls. However, in general, the prevalence of obesity among the urban boys was higher than in the urban girls and the change between 2013 and 2016 was larger in the urban boys than in the urban girls. Larger increase of obesity for boys than girls has been reported in preschool age children [28] and in older children [19, 26]. In the analysis of changes within the cohort, we found that the difference occurs mainly because more obese urban boys continued to remain obese and that normal urban boys became obese more often than urban girls. In the rural area, there was no statistically significant difference between boys and girls in prevalence of obesity but the prevalence of overweight among the boys was statistically significantly higher than for the girls. It thus seems that boys are more at risk to become overweight or obese than girls.

In this study, the percentage of children remaining in already established overweight was 49.5% and 41.9% among urban boys and urban girls, respectively. This is much lower than the 90% for boys and 66.7% for girls seen in urban Indonesian children aged 6–8 year old [34]. The percentage of children remaining in obesity was less than 40% in the present study compared to 100% in the Indonesian children.

Increasing prevalence of obesity was mainly seen in the youngest children aged 3 years in 2013, while there was a decrease among older children. Literature has mentioned that critical periods for obesity development include the prenatal period, the period of adiposity rebound occurring between 4 and 7 years of age [35, 36], and the period of adolescence [36, 37]. The period of adiposity rebound with its expected BMI increase could be a risk stage for earlier and greater increase of BMI. The child sensitivity during this period should be taken into consideration as an explanation for the difference in obesity pattern between the children aged 3 years at the start of study and the older age children in the study.

This is the first longitudinal cohort study on preschool overweight and obesity in Vietnam. Reasonably large groups of children in one urban and one rural setting were followed for three years. Comparatively few children dropped out. The system used for data management and control was well established since earlier studies in the same contexts. We consider the internal validity as good.

The two sites studied do not represent the general Vietnamese population as Hanoi is quite different from Ho Chi Minh City and other provinces in various respects. External validity, that is the generalizability outside the investigated areas, in specific aspects can be based on theory and detailed studies of contexts [38]. A specific problem in this type of follow-up is that the elapsed time for the study is the same as the age increase for the participants. Therefore, we cannot conclude how the changes observed are actually effects of changed influences over time and the parallel changing age of the children.

## Conclusions

There is, already at preschool age, a substantial number of overweight children, and the prevalence continue to increase with increasing ages. Both the prevalence and the increase of prevalence were larger for urban children than for rural. The prevalence of obesity is stable with some tendencies to decrease, similarly for urban and rural children. For overweight, there were no large differences between boys and girls. However, boys were more likely to develop obesity than girls. Prevention and intervention programs need to start at early preschool age and actions in urban areas deserve priority.

## Abbreviations

BMI: Body Mass Index
HDSS: Health and Demographic Surveillance Sites
IOTF: International Obesity Task Force
Ob: Obesity
Ow: Overweight

## References

[1] Magarey AM, Daniels LA, Boulton TJ (2001). Prevalence of overweight and obesity in Australian children and adolescents: reassessment of 1985 and 1995 data against new standard international definitions. Med J Aust.

[2] Kautiainen K, Rimpela A, Vikat A, Virtanen SM (2002). Secular trends in overweight and obesity among Finnish adolescents in 1977-1999. Int J Obes.

[3] Wang Y, Lobstein T (2006). Worldwide trends in childhood overweight and obesity. Int J Pediatr Obes.

[4] Ebbeling CB, Pawlak DB, Ludwig DS (2002). Childhood obesity: public-health crisis, common sense cure. Lancet.

[5] von Hippel PT, Nahhas RW (2013). Extending the history of child obesity in the United States: the Fels longitudinal study, birth years 1930 to 1993. Obesity (Silver Spring).

[6] Wabitsch M, Moss A, Kromeyer-Hauschild K (2014). Unexpected plateauing of childhood obesity rates in developed countries. BMC Med.

[7] Ogden CL, Carrol M, Curtin LR, Lamb MM, Flegal KM (2010). Prevalence of high Body Mass Index in US children and adolescents, 2007-2008. JAMA.

[8] O'Dea JA, Nguyen Hoang TDH, Dibley MJ (2011). Plateau in obesity and overweight in a cross sectional study of low, middle and high socioeconomic status schoolchildren between 2004 and 2009. Int J Public Health.

[9] Mazur A, Klimek K (2014). Ten-year secular trend of overweight and obesity in school children in south-eastern Poland. Annal of Argicultural and Environmental Medicine.

[10] Olds T, Maher C, Zumin S, Peneau S, Lioret S, Castetbon K (2011). Evidence that the prevalence of childhood overweight is plateauing: data from nine countries. Int J Pediatr Obes.

[11] Lazzeri G (2015). Trend in overweight and obesity prevalence in Tuscan schoolchildren (2002-2012). Public Health Nutr.

[12] de Onis M, Blossner M, Borghi E (2010). Global prevalence and trends of overweight and obesity among preschool children. Am J Clin Nutr.

[13] Popkin BM, Gordon-Larsen P (2004). The nutrition transition: worldwide obesity dynamics and their determinants. Int J Obes Relat Metab Disord.

[14] Gupta N, Goel K, Shah P, Misra A (2012). Chilhood obesity in developing countries: epidemiology, determinants, and prevention. Endocr Rev.

[15] Nguyen CK, Ha HK (2008). Double burden of malnutrition: the Vietnamese perspective. Asia Pac J Clin Nutr.

[16] General Statistics Office. Socio-economic situation of Vietnam from 2001–2010. Part I -Overview of Socio-economic situation in Vietnam from 2001–2010 Hanoi: Statistical publisher; 2011. p. 8–9.

[17] Trang NHHD, Hong TK, Dibley MJ (2012). Cohort profile: Ho Chi Minh City youth cohort—changes in diet, physical activity, sedentary behaviour and relationship with overweight/obesity in adolescents. BMJ Open.

[18] WHO. Vietnam - Child obesity 2014 [cited 2016 February 20] Available from: http://www.indexmundi.com/facts/vietnam/child-obesity. Accessed 20 Feb 2016.

[19] Hong TK, Trang NHHD, Dibley MJ (2013). Changes in adiposity indicators of Ho Chi Minh City adolescents in a 5-year prospective cohort study. Int J Obes.

[20] Do LM, Tran KT, Eriksson B, Petzold M, Nguyen CT, Ascher H (2015). Preschool overweight and obesity in urban and rural Vietnam: Differences in prevalence and associated factors. Global Health Act.

[21] Tran KT, Eriksson B, Nguyen TC, Horby P, Bondjers G, Petzold M (2012). DodaLab - an urban health and demographic surveillance site, the first three years in Hanoi, Vietnam. Scand J Public Health.

[22] Chuc TN, Diwan VK (2003). FilaBavi, a demographic surveillance site, an epidemiological field laboratory in Vietnam. Scand J Public Health.

[23] Cole TJ, Bellizzi MC, Flegal KM, Dietz WH (2000). Establishing a standard definition for child overweight and obesity worldwide: international survey. BMJ.

[24] Le Nguyen BK, Le Thi H, Nguyen Do VA, Tran Thuy N, Nguyen Huu C, Do TT (2013). Double burden of undernutrition and overnutrition in Vietnam in 2011: results of the SEANUTS study in 0·5–11-year-old children. Br J Nutr.

[25] National Institute of Nutrition, UNICEF. General Nutrition Survey (2009). Part D -findings and discussions. Hanoi: Medical Publising House.

[26] Dang VC, Day SR, Selwyn B, Maldonado MY, Nguyen CK, Le DT (2010). Initiating BMI prevalence studies in Vietnamese children: changes in a transitional economy. Asia Pac J Clin Nutr.

[27] Davison KK, Birch LL (2001). Childhood overweight: a contextual model and recommendations for future research. Obes Rev.

[28] Dieu HTT, Dibley MJ, Sibbritt D, Hanh TTM (2008). Trends in overweight and obesity in pre-school children in urban areas of ho chi Minh City, Vietnam, from 2002 to 2005. Public Health Nutr.

[29] Thuan V, Hieu Q. Thu nhập bình quân người dân TP HCM hơn 5.500 USD 2015 [cited 2016 April 25] Available from: http://news.zing.vn/thu-nhap-binh-quan-nguoi-dan-tp-hcm-hon-5500-usd-post589744.html. Accessed 25 Apr 2016.

[30] Ngoc Yen. Thu nhập bình quân đầu người của Hà Nội khoảng 3.600 USD 2015 [cited 2016 April 25]. Available from: http://cand.com.vn/Su-kien-Binh-luan-thoi-su/Thu-nhap-binh-quan-dau-nguoi-cua-Ha-Noi-khoang-3-600-uSd-374700/. Accessed 25 Apr 2016.

[31] Fernandes MM (2013). A national evaluation of the impact of state policies on competitive foods in schools. J Sch Health.

[32] Iannotti RJ, Wang J (2013). Trends in physical activity, sedentary behavior, diet, and BMI among US adolescents, 2001-2009. Pediatics.

[33] Agency for Healthcare Research and Quality. Childhood Obesity Prevention Programs: Comparative Effectiveness Review and Meta-Analysis. Comparative Effectiveness Review No. 115. 2013 [cited 2016 June 12] Available from: https://effectivehealthcare.ahrq.gov/ehc/products/330/1523/obesity-child-executive-130610.pdf. Accessed 12 June 2016. 23865092

[34] Julia M, van Weissenbruch MM, Prawirohartono EP, Surjono A (2008). Delemarre-van de Waal HA. Tracking for underweight, overweight and obesity from childhood to adolescents: a 5-year follow-up study in urban Indonesian children. Horm Res.

[35] Dietz WH. Period of Risk in Childhood for the Development of Adult Obesity-What Do We Need to Learn? J Nutr. 1997;127(9):1884s–6s. 10.1093/jn/127.9.1884S 9278575

[36] WHO. Obesity: Preventing and managing the global epidemic. Report of a WHO Consultation. Geneva, Switzerland. (WHO technical report series 894). 2000;894:i-xii, 1–253. 11234459

[37] Dietz WH (1994). Critical periods in childhood for the development of obesity. Am J Clin Nutr.

[38] Smaling A. Inductive, analogical, and communicative generalization. International Journal of Qualitative Methods. 2003;2(1).

